# Peptides from General By-Products: Unveiling Their Potential Biological Activities in Human Health

**DOI:** 10.3390/molecules30244821

**Published:** 2025-12-18

**Authors:** Alejandra Colón-Sandoval, Laura A. Contreras-Angulo, Luis A. Cabanillas-Bojórquez, Erick Paul Gutiérrez-Grijalva, Josefina León-Félix, Nayely Leyva-López, Leticia Xochitl López-Martínez, Miriam D. García-Cebreros, José Basilio Heredia

**Affiliations:** 1 Centro de Investigación en Alimentación y Desarrollo, A.C. Nutraceutical and Functional Foods, Carretera a Eldorado Km. 5.5, Col. Campo El Diez, Culiacán 80110, Sinaloa, Mexico; acolon223@estudiantes.ciad.mx (A.C.-S.); lcontreras@ciad.mx (L.A.C.-A.); ljosefina@ciad.mx (J.L.-F.); nayely.leyva@ciad.mx (N.L.-L.); 2 Posdoc SECIHTI-Centro de Investigación en Alimentación y Desarrollo, A.C. Nutraceutical and Functional Foods, Carretera a Eldorado Km. 5.5, Col. Campo El Diez, Culiacán 80110, Sinaloa, Mexico; luis.cabanillas@ciad.mx; 3 Laboratory of Functional Foods and Nutraceuticals, Researchers for Mexico Program SECIHTI-Centro de Investigación en Alimentación y Desarrollo, A.C. Nutraceutical and Functional Foods, Carretera a Eldorado Km. 5.5, Col. Campo El Diez, Culiacán 80110, Sinaloa, Mexico; erick.gutierrez@ciad.mx; 4 SECIHTI-CIAD, Antioxidants and Functional Foods, Carr. Gustavo Enrique Astiazarán Rosas 46, Col. La Victoria, Hermosillo CP 83304, Sonora, Mexico; 5 College of Nutrition and Gastronomy Sciences, Universidad Autónoma de Sinaloa, Ave. Cedros y Calle Sauces s/n, Fracc. Los Fresnos, Culiacán 80034, Sinaloa, Mexico; miriamgarcia.uacng@uas.edu.mx

**Keywords:** animal by-products, plant by-products, marine by-products, waste, hydrolysis, fermentation, bioactive peptides

## Abstract

Peptides are short amino acid chains that can be released from proteins through hydrolysis or fermentation, exhibiting various biological activities, including antioxidant, antihypertensive, anti-inflammatory, and anticancer effects. Generally, these compounds are extracted from food products. However, to maximize resource utilization under the premise of sustainability in favor of a circular economy, there is an interesting approach to obtaining peptides from sub-utilized parts and non-food products of food production and processing, based on the grade at which they can be valued. These by-products may contain large quantities of protein that can be utilized. Although some may have a low protein content, they stand out for the quality and proportion of their amino acids, which provide properties with functional applications. This revision approaches some of the most recent reports on isolated peptides from residues, by-products, or underutilized parts from plant, animal, or sea origin; the conventional methods and alternative technologies to isolate them from the origin matrixes; the methods to purify and identify them, the biological activities they perform, as well as a brief description of the application fields of these compounds and the challenges that their application faces in biomedicine and the food industry.

## 1. Introduction

Amino acids linked by peptide bonds form peptides, short chains ranging from two to fifty amino acids. Conversely, oligopeptides consist of chains between ten and fifteen amino acids, while polypeptides comprise chains with more than fifteen amino acids [1].

Peptide studies have been extensively explored, and bioactive peptides offer various health-promoting benefits that extend beyond basic nutritional needs. According to the order and frequency of biological functions reported, they can function as food enhancers, food additives, as well as hypoglycemic, antibacterial, antihypertensive, antioxidant, and anti-inflammatory agents. Additionally, they can act as immunomodulators, antimicrobials, antiproliferative, and hypocholesterolemic agents [1,2].

Regarding their structure and function, there is numerous scientific evidence that, besides the molecular weight, the peptides’ functions are closely related to their amino acid composition. In particular cases, the biological functions are guided by a defined sequence of amino acids, while in others, they depend on the relative proportion of a specific amino acid or a group of amino acids [3]. For example, the amino acid composition, hydrophobic properties, weight, peptide length, and the type of residual C- and N-terminal all influence the functional properties and biological activities of peptides [4]. In peptides with low molecular weights, the folding of space structures is restricted, which exposes the amino acids completely and enhances their functionality [5]. Therefore, the general composition of amino acids, their physical and chemical properties, and the structure of peptides play a crucial role in their various biological activities [6].

Given the vast potential of these molecules, the residues and by-products of the food industry, agriculture, or non-food underutilized parts can be a rich source of bioactive compounds. The protein fraction has garnered considerable attention from researchers because, in some cases, the proportions of these residues are relatively high. The quality of amino acids from these proteins may be valued for others because they can produce functional peptides in the food industry and biomedical applications [7].

A peptide is found in an inactive form within the original protein and must be isolated through conventional processes, such as chemical and enzymatic hydrolysis, fermentation, or germination [8]. Enzymatic hydrolysis is the most used process due to the specificity of proteolytic enzymes. Various factors, including proteases, hydrolysis duration, the enzyme–substrate (E/S) ratio, and the type of protein substrate, influence biological activities [9]. These isolation processes may also utilize alternative technologies, such as previous treatments to hydrolysis, including microwave-assisted extraction (MAE), ultrasound-assisted extraction (UAE), ohmic heating, pulsed electric fields (PEFs), hydrolysis in subcritical water (SCW), and high hydrostatic pressure (HHP) [10]. These technologies, as well as purification methods such as ultrafiltration, ion exchange chromatography, electrophoresis, and reversed-phase high-performance liquid chromatography (RP-HPLC), identification methods including ESI-MS, MALDI-TOF, LC-MS/MS, and LC-Q-TOF, and in silico peptide analysis, are methods that have been included in this revision.

The importance of this research lies in the peptides’ capacity to engage with and intervene in biological processes, offering a natural alternative for modulating human health. Therefore, the valorization of protein-rich agro-industrial, vegetable, and marine subproducts and residues not only solves a waste management issue but also drives the circular economy by transforming underutilized materials into high-value compounds. Consequently, this review aims to consolidate the most recent knowledge on these strategies and lay the groundwork for the development of future sustainable nutraceuticals and pharmacological agents.

To establish the scope and define the article selection methodology of this narrative review, an exhaustive bibliographic search was conducted across the PubMed and Google Scholar databases. The search focused on identifying the most recent publications, preferably those not exceeding 10 years in age, concerning bioactive peptides originating particularly from subproducts and waste derived from agro-industries, agriculture, and marine sources. The search terms utilized and their combinations included: peptides, hydrolysates, by-products/residues, animal, plant, marine, valorization, and extraction, which yielded an initial search record of 18,000 results. Inclusion criteria focused on original research articles and comprehensive reviews, specifically those detailing the extraction, purification, identification, and in vitro or in vivo biological activity of peptides from agro-industrial subproducts or waste. Exclusion criteria dismissed documents older than 10 years (unless their relevance was exceptional), reports of peptides sourced from consumable (non-residual) parts of plants, animals, or marine organisms, articles where the full text was unavailable, non-English language articles, and non-academic publications such as patents, conference abstracts/posters, or unpublished theses. This selection process resulted in a comprehensive review of 193 articles, which are summarized in this document.

## 2. Conventional Methods for Obtaining Peptides

### 2.1. Chemical Hydrolysis

Chemical hydrolysis is a method that uses chemical compounds or techniques to obtain bioactive peptides. In this method, two types of hydrolysis are distinguished: acidic hydrolysis, where acids are commonly added, and alkaline hydrolysis, where bases such as sodium hydroxide (NaOH) are frequently used. Together with high temperatures, these reagents can induce chemical reactions in proteins, leading to peptide cleavage. However, chemical hydrolysis is unsuitable for obtaining bioactive peptides due to several limiting factors. It is a difficult-to-control process, which can generate low yields and cause the denaturation of amino acids [11].

Acid hydrolysis is a crucial chemical modification that alters the structure and functional properties of peptides. The most common types of diluted acid are hydrochloric acid (HCl) and sulfuric acid (H_2_SO_4_). However, nitric acid (HNO_3_), phosphoric acid (H_3_PO_4_), and other acids have also been investigated [12]. However, acid hydrolysis generally requires high temperature, which could decrease essential amino acids from the protein structure. On the other hand, alkaline hydrolysis results in poor functionality and low nutritional value. In this sense, chemical hydrolysis can cause the cleavage of peptide bonds to obtain peptides; however, various studies have suggested that it is an unsafe and environmentally hostile method, which is why it is typically used mainly for industrial production [13,14,15].

### 2.2. Enzymatic Hydrolysis

There are multiple methods for obtaining bioactive peptides from by-products, where enzymatic hydrolysis is the most common and effective technique. This method involves incorporating commercial enzymes that are responsible for cleaving the peptide bonds established in the protein, thereby releasing peptides with biological activity [16]. The preferred commercial enzymes are prepared from bacterial and fungal origins, such as alcalase, neutrase, and flavourzyme, as well as those from plant origins, including papain and bromelain. Other proteases are the digestive enzymes from the gastrointestinal tract of animal origin, such as pepsin, trypsin, and chymotrypsin [17]. Likewise, each enzyme has different hydrolysis conditions, such as pH, extraction temperature, time, enzyme concentration, and water-to-material ratio, which offer advantages over chemical treatments. Exogenous enzymes can make the hydrolytic process more controllable and reproducible, as they exhibit substrate specificity, facilitating the obtaining, manipulation, and use of peptides [18].

For enzymatic hydrolysis to occur, it is essential that the enzyme first binds to the substrate and then proceeds with enzymatic catalysis. For this, the enzyme has specific active sites that contain residues that form temporary bonds with the substrate and residues that catalyze the reaction with the substrate, thereby forming binding sites and catalytic sites within the enzyme–substrate complex through hydrogen bonds, hydrophobic interactions, or Van der Waals forces. Finally, protein hydrolysis is performed in a specific conformation, cleaving the established peptide bonds in proteins and releasing bioactive peptides [19].

Likewise, enzymatic hydrolysis does not usually generate secondary products, it does not use synthetic chemicals, generating a more environmentally friendly concept, and it is one of the most used methods in the industry, obtaining higher yields of peptides and with better quality; the enzymes are easy to inactivate, and in general, the process is simple to use [20,21].

### 2.3. Fermentation

Microbial fermentation is a process in which microorganisms break down complex organic matter into smaller molecules. It has traditionally been used to preserve food; however, in recent times, it is also used to improve components from different materials, as well as to reduce antinutritional factors such as phytic acid, proteinase inhibitors, urease, and oxalic acid, among other compounds [22]. The fermentation process can be divided into two principal technologies: solid-state fermentation (SSF) and submerged fermentation (SF). In this process, microorganism growth occurs in the absence or presence of free water from the food matrix [23]. SF is one of the most studied methods for obtaining bioactive compounds from microorganisms. The method can be applied on a large scale, and the conditions can positively impact the diffusion of microorganisms within the food matrix, thereby enhancing the separation of target compounds [24]. SF can be performed in three ways: batch, fed-batch, and continuous fermentation, where nutrients are added at different periods to meet the microorganisms’ growth requirements [25,26].

On the other hand, SSF is used to obtain products rich in mycoprotein, as the fermented microorganism (primarily fungi) constitutes a substrate for solid fermented products, in contrast to SF, where the products need to be separated from the liquid matrix [25,26]. In the fermentation process, the microorganisms studied are primarily bacteria; however, fungi are extensively used in SSF, as some fungi require less water to grow, in contrast to yeast and bacteria. Nevertheless, different authors reported that some fungi could perform better in SF, so the microorganisms studied are related to the interested product [26]. These microorganisms produce enzymes called cell envelope proteinases, which, in combination with other intra- or extracellular proteases, break down proteins and release bioactive compounds into the culture medium, such as peptides and free amino acids. These compounds are typically separated by centrifugation and subsequent peptide analysis [23,26,27]. Authors reported that for an optimal fermentation process, several key factors, such as the selection of microorganism, substrate particle size, fermentation conditions (pH, agitation, temperature, aeration, and fermentation time), and bioreactor design, need to be studied [23,25,26,28,29].

Lactic acid bacteria have been utilized as microorganisms with several advantages over yeast, as they are GRAS (generally recognized as safe) bacteria with potential applications as starter cultures in food [23]. Among these lactic acid bacteria, *Lactobacillus* are the principal bacteria studied to obtain bioactive compounds from milk, eggs, and other sources [30]. It has been reported that *Lactobacillus* produces lactic acid, aroma compounds, bacteriocins, and several enzymes that contribute to reducing the growth of other microorganisms. Additionally, *Lactobacillus* bacteria can produce secondary metabolites from the protein source used in the fermentation process [31]. On the other hand, yeast has been used as a co-culture with lactic acid bacteria due to the expression of various enzymes by the yeast, such as carboxypeptidases and aminopeptidases, which enhance the production of bioactive peptides [32]. In fungal fermentation, it has been reported that morphology is related to the production and secretion of proteins, which may be associated with the production of peptides. The productivity of peptides may be affected [26]. Therefore, microbial fermentation has been studied to obtain bioactive peptides from various sources (Table 1) using different microorganisms, including yeast [32], bacteria [23,27,33], and fungi [25,26,34].

### 2.4. Physical and Mechanical Methods

Direct extraction of peptides from the food matrix is based on physical procedures, where shear forces disrupt cells and release proteins, denaturing them into peptide fractions. Among these methods are mechanical crushing, colloid milling, homogenizing, and freeze–thaw [35,36]. Physical techniques to obtain proteins are scalable industrially because the equipment and procedures are economically and easily replicable. However, in recent years, these methods have been less used due to the low purification efficiency [35,37].

**Table 1:** Microorganisms studied for peptides derived from food fermentation.

Type	Microorganism	Fermentation	Food Matrix	Reference
Bacteria	*Bacillus*, *Bifidobacterium*, *Lactobacillus*, *Pediococcus*, *Streptococcus*, *Weissella*	Submerged Fermentation	Apple, carp head clam, crab shell, fish, meat, milk, sardinella muscle, sea bass, shrimp paste	[23,24,36,38,39,40]
	*Bifidobacterium*, *Cordyceps*, *Lactobacillus*, *Lactococcus*, *Pediococcus*	Solid State Fermentation	Cassava, cottonseed meal, dehusked barley, jatropha seed, lentil, maize, rice, oat, red sorghum, shrimp, soybean, wheat bran.	[25,34,41,42,43]
Fungi	*Aspergillus*, *Candida*, *Cordyceps*, *Galactomyces*, *Rhizopus*	Submerged Fermentation	Barley, bean, chokeberry, grapefruit, milk, plum, turbot skin	[23,24,44,45,46]
	*Aspergillus*, *Lentinus*, *Mucor*, *Rhizopus*, *Thamnidium*, *Trichoderma*	Solid State Fermentation	Brewers spent grain, brown rice, chickpea, colored quinoa, lupin, maize, moringa, oat, olive, orange peel, peanut, pineapple peels, quinoa, soybean, wheat,	[25,34,47,48]
Yeast	*Kluyveromyces*, *Monascus*, *Pichia*, *Saccharomyces*, *Torulaspora*	Submerged Fermentation	Apple, milk, porcine liver	[44,49]
	*Pichia kudriavzevii*, *Saccharomyces*, *Yarrowia lipolytica*	Solid State Fermentation	Guar and copra meal, nut oil cake, quinoa, soja, rye bread, wheat.	[34,50,51,52,53]

## 3. Emerging Technologies for Peptide Production

**Figure 1:**
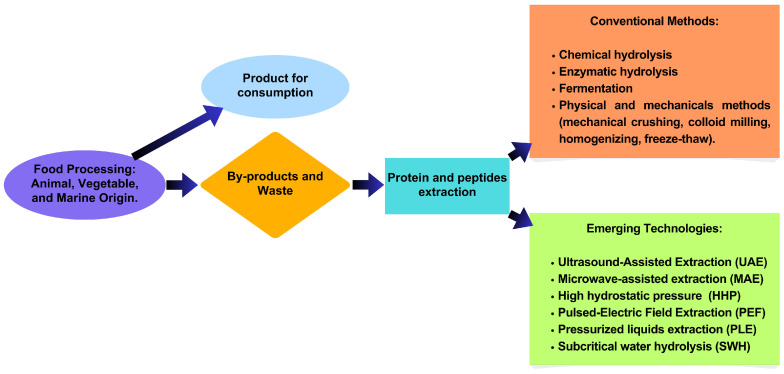
Peptide extraction from by-products and waste.

Biochemical hydrolysis is the most common method used to improve protein extraction and protein hydrolysis, thereby obtaining hydrolysates or bioactive peptides. However, emerging technologies such as Ultrasound-Assisted Extraction (UAE) or sonication extraction, Microwave-Assisted extraction (MAE), High Hydrostatic Pressure (HHP), Subcritical Extraction Water (SEW), and Pulsed Electric Field (PEF) (Figure 1) are being developed to reach higher yields of hydrolysates or peptides and to lower costs. However, studies on the effect of food by-products on protein production, hydrolysates, or peptides remain limited (Table 2).

### 3.1. Ultrasound-Assisted Extraction (UAE)

UAE implies the use of ultrasound ranging from 20 kHz to 2000 kHz. The mechanical effect of acoustic cavitation involves the formation, growth, and collapse of bubbles; as a consequence, a lot of energy is produced, causing extreme changes in temperature (up to 4700 °C) and pressure (100 MPa) within bubbles during extraction, causing liquid solutes to leach at speeds of 280 m/s [54]. Using ultrasound as a pre-treatment for hydrolysis may alter the conformation and structure of proteins, thereby shifting hydrophilic interactions or hydrogen bonds and affecting the proteins’ tertiary and quaternary structures. Along with the mechanical and physical impacts, ultrasound may enhance the exposure of hydrolysis sites, making them more vulnerable to the action of proteinases and accelerating the breakdown of proteins by proteinases, thereby intensifying hydrolysis [55].

### 3.2. Microwave-Assisted Extraction (MAE)

MAE is a heat source generated by electromagnetic waves (with a frequency range of 300 MHz to 300 GHz) from a cavity magnetron. The tissues and cell walls interact with the emitted radiation waves without direct contact with the sample, which can provide more effective heating, accelerate energy transfer, and reduce the thermal gradient. Compounds such as protein, hydrolysates, and peptides have been separated using this technology. Microwave heating has been employed as a pretreatment step before enzymatic hydrolysis, and it has been found that this method enhances the degree of hydrolysis and protein solubility, thereby improving the accessibility and susceptibility of bonds to enzymes [56,57].

### 3.3. High Hydrostatic Pressure (HHP)

HHP has recently been employed to hydrolyze several food by-product proteins, in conjunction with enzymatic hydrolysis (HHP-assisted enzymatic hydrolysis), to enhance the release of hydrolysates or bioactive peptides. HHP is a non-thermal treatment that uses pressures between 100 and 1000 MPa, which can alter the extension and conformation of protein sequences, facilitating proteolysis and allowing enzymes to cleave peptide bonds at new sites, thereby generating bioactive peptides [58].

### 3.4. Pulsed-Electric Field Extraction (PEF)

PEF extracts intracellular content through electroporation by breaking down cell membranes with high voltages and brief electric pulses. PEF is affected by factors such as electrical strength, pulse characteristics, cell characteristics, and the capabilities of the equipment [59,60]. These factors must be optimized to extract proteins, hydrolysates, and peptides from by-products of plant, animal, and marine origin for the intended results. PEF offers benefits, including improving mass transfer without adverse consequences and being suitable for heat-sensitive foods without the use of hazardous solvents [61].

### 3.5. Pressurized Liquid Extraction (PLE)

The compounds of interest are extracted by PLE using pressures and temperatures ranging from 35 to 200 bar and 50 to 200 °C, respectively. The high temperature lowers surface tension and liquid viscosity while increasing solubility and mass transfer rate. The solvent may rise above its typical boiling point while still in a fluid state due to increased pressure. These circumstances facilitate the mass transfer of analytes and the penetration of solvents into the sample matrix [62]. PLE is frequently carried out using solvents such as ethanol or water [63,64]. PLE has been little used to produce hydrolysates or peptides from food by-products. The highest extraction yield was obtained using 70% (*v*/*v*) ethanol as the extracting solvent, and 120 °C for 3 min (9 mg protein/g pomegranate peel) was achieved [63]. Under optimal conditions, 22% EtOH, 129 °C, and 7 min, for Mexican lime peels, and 75% EtOH, 134 °C, and 11 min, for Spanish lime peels, the extraction yields were 4.5 and 5.0 g protein/100 g dried peel, respectively. Proteins extracted by PLE were hydrolyzed with thermolysin, and the 98 peptides released were identified by UHPLC-MS/MS [64].

### 3.6. Subcritical Water Hydrolysis (SWH)

SWH uses water as a solvent in a sub-critical state; in this state, water can extract non-polar, polar, and ionic compounds, leading to hydrolysis and the breakdown of the protein structure, consequently releasing hydrolysates or peptides. Amino acid extraction is possible if the extraction duration is extended [65]. Vapor pressure, polarity, and molecular weight all affect the solubility of proteins during subcritical extraction [66].

**Table 2:** Extraction methods for bioactive hydrolysates and peptides derived from food by-products.

By-Product	Treatment Type	Extraction Conditions	Protein, Hydrolysate, or Peptide Fraction	Outcomes	Reference
**Ultrasound-assisted extraction (UAE)**					
Carp scales	Distilled water	Temperature: 60 °C Extraction time: 1, 3, and 5 h	Gelatin with high protein content (84.15 ~ 91.85%)	Protein extraction yield: 46.67%	[67]
Slurry from fabrication of milk soy	Distilled water	20 kHz, 400 W for 0, 0.5, 1, 5, and 15 min.	Size of the products was not defeminated	Protein extraction yield: 60%	[68]
Jackfruit leaves	0.5 M NaCl	10, 15, or 20 min at 25 °C and 42 kHz.	70 Da to 14 kDa	Protein extraction yield: 96.3 mg/g	[69]
Bovine bones	Pepsin, 76.25 × 10^4^ U/g (double hydrolysis)	power of 413.90 W/L, pH 9, for 2.01 h.	Size of the products was not defeminated	Yield of the peptide: 21.04%.	[70]
**Microwave-assisted extraction (MAE)**					
Trout by-products	Hydrolyzed with alcalase 0.5, 1.7, and 3.0% (*w*/*v*), respectively, for 3, 5, and 15 min	For the microwave heating treatments (1200 W, 20% power with 50% duty cycle at 50–55 °C)	Hydrophobic amino acids and histidine	As E:S and hydrolysis time increased, the hydrolysis degree increased, ranging from 11% to 23%.	[56]
Jackfruit leaves	0.5 M NaCl	Microwave power at 1200 W, applying pulses of 30 s for 4 min	70 to 14 kDa	Protein extraction yield: 87.6 ± 0.8 mg/g	[69]
Sesame bran	Deionized water, 1.94 AU/100 g of alcalase	Microwave power at 750 W, 49 °C, for 8 min	22–30 kDa	Extraction yield: 43.8 to 61.6%	[71]
Coffee green beans	Alkaline-acid extraction	Microwave power at 900 W, 50% capacity for 240 s.	Asp, Phe, Glu, Lys	The combination with alkali extraction increases the protein extraction by 77%.	[57]
**High hydrostatic pressure (HHP**)					
Jackfruit leaves	0.5 M NaCl	100, 200, or 300 MPa, for periods of 10, 15, and 20 min	70 Da to 14 kDa	Protein extraction yield: 147.3 mg/g	[69]
Bighead carp bones	1 M NaOH, hydrolyzed with alcalase (3 mL/100 mL)	250 MPa for 35 min.	Protein content: 5.7 mg/mL All essential amino acids	Soluble protein content increased with increasing pressure and holding time. The process accelerated hydrolysis and facilitated the release of free amino acids.	[72]
**Pulsed-Electric Field Extraction (PEF)**					
Fishbone	Pepsin 1%	Electric field strength 22.79 kV/cm and pulse number 9	Collagen	3.875 mg/mL	[73]
Waste breast Muscle from chicken	Combined with mechanical pressing	37.6 ± 4.69 J/g	Size of the products was not defeminated	Extraction of 12% of the initial biomass with the protein content of 78 mg/mL from the liquid fraction.	[65]
**Subcritical water hydrolysis (SWH)**					
Tuna skin		Different temperature (150–300 °C) at pressure (50–100 bar) and reaction time 5 min	Peptides of molecular size less than 600 Da and some free amino acids	The degree of hydrolysis was highest at 250 °C	[74]
Porcine placenta	Assisted by hydrolysis with trypsin	Processing at 37.5 MPa and 200 °C	Peptides of 626 Da	SCW pre-treatment followed by trypsin produced peptides of 626 Da SCW alone produced peptides of 10 kDa	[75]

## 4. Sources of Peptide Extraction

Due to the significant relevance that bioactive peptides have as a functional ingredient, techniques have been developed to search for new protein sources from various origins, including animal, plant, and marine. Multiple studies have been conducted in the search for bioactive peptides, primarily from food sources consumed by humans, utilizing residues and by-products derived from these nutrients, including those from sources such as leaves, stems, and roots of certain vegetables. The residue generated by the agro-industries may be a rich source of bioactive components of great nutraceutical and functional value. Recently, due to the processing of different foods, a significant amount of subproducts and residues is generated, which, in general, contain a substantial amount of protein that can be used to produce hydrolyzed and peptide products with potential biological activities [76].

### 4.1. Peptides from Animal By-Products

Since ancient times, the primary source of protein has been of animal origin, not only for its nutritional contribution but also as an essential source of peptides, which can have different biological functions. The primary sources of these proteins and peptides are milk, eggs, and meat [13,77]. Derived from the consumption and processing of food, particularly from proteins of animal origin, numerous by-products are generated, which have proven to be a significant source of peptides with bioactive properties (Table 3) [78].

In this sense, Dibdiakova et al. [79] obtained bioactive peptides from chicken by-products through different separation methods such as microfiltration, ultrafiltration, nanofiltration, and reverse osmosis, finding that ultrafiltration was the most efficient method in the concentration of low molecular weight peptides and that it presented a significant effect in the inhibition of DPP-IV (IC_50_ 0.75 mg/mL) target protein in the treatment of type 2 diabetes. On the other hand, Lee et al. [80] reported that gelatin hydrolysates obtained from duck skin, a by-product in farms, exhibited antioxidant and inhibitory activity against the angiotensin-converting enzyme (ACE). Likewise, from this investigation, an antioxidant peptide was isolated and identified, which had a molecular weight of 941.43 Da and consisted of a chain of seven amino acids (His-Thr-Val-Gln-Cys-Met-Phe-Gln); they also found that this peptide inhibits the formation of reactive oxygen species, activates antioxidant enzymes, exerts a protective effect against alcohol-induced damage in liver cells, and according to their results could be acting as a regulator in the expression of genes related to apoptosis [81].

**Table 3:** Peptides from animal by-products.

By-Product	Process	Peptide/Sequence	Bioactivity	Reference
Egg yolk protein	Hydrolysis with proteinase from *Cucurbita ficifolia*	LAPSLPGKPKPD, RASDPLLSV, RNDDLNYIQ, AGTTCLFTPLALPYDYSH.	Antioxidant activity, inhibition of α-glucosidase and DPP-IV.	[82]
Egg yolk protein	Hydrolysis with pepsin	YINQMPQKSRE, YINQMPQKSREA, VTGRFAGHPAAQ, YIEAVNKVSPRAGQF.	Antioxidant activity and inhibits DPP-IV, ACE, and α-glucosidase.	[83]
Chicken intestine	Autolytic digestion	ARIYH, LRKGNLE, RVWCP.	ACE inhibitory activity	[84]
Ground turkey head	Hydrolysis with enzyme cocktail (alcalase, flavoenzyme, trypsin)	Collagen peptide of low molecular weight (555.26–2093.74 Da).	Anti-inflammatory and anticholesterolemic.	[85]
Sheep abomasum	Hydrolysis with neutrase, alcalase, and papain	LEDGLK, IDDVLK.	Antioxidant activity	[86]
Bovine hair	Hydrolysis with alcalase	CERPTCCEHS	Antioxidant activity, inhibits hemolysis and lipid peroxidation, protects against DNA damage	[87]
Duck liver	Fermentation with *Bacillus subtilis*	MYGAVTPVK, NWEKIR, APGIIP, RWWQLR.	High antioxidant activity reduces intracellular oxidative damage	[88]
Mechanical chicken deboning residue	Hydrolysis with the proteases flavourzyme and corolase	Dipeptides with leucine or isoleucine at the N-terminal	Inhibits DPP-IV and increases glucose uptake.	[89]
Dry-cured ham by-products	Hydrolysis with acid and heat	Not specified (212 identified)	ACE, endothelin-converting enzyme (ECE), platelet-activating factor-acetylhydrolase (PAF-AH), and DPP-IV inhibitory activity	[90]
Milk permeates	Solid-phase	Sequences derive from α and β caseins, lactoperoxidase, lipoprotein lipase, glycosylation-dependent cell adhesion molecule 1, and polymeric immunoglobulin receptor.	Increase antioxidant enzymes and inhibit oxidative stress by activating the Keap1/Nrf2 axis.	[91]
Combs and wattles of chicken	Hydrolysis with alcalase	APGLPGPR, Piro-GPPGPT, FPGPPGP.	Inhibitory activity of ACE	[92]
Bovine hemoglobin	Pig pepsin	TKAVEHLDDLPGALSELSDLHAHKLRVDPVNFKLLSHSLL; LDDLPGALSELSDLHAHKLRVDPVNFKLLSHSL, KLLSHSL, LLSHSL.	Antimicrobial and antihypertensive	[93]
Bovine hemoglobin	Pepsin	Neokyotophin (NKT) peptide	Antimicrobial activity	[94]

### 4.2. Peptides from Vegetable By-Products

Vegetable foods, including cereals, legumes, fruits, and vegetables, are a source of bioactive peptides and are recognized for their nutraceutical profiles and acceptability advantages due to their origin. They also serve as a readily available and affordable source of protein. Protein hydrolysates and bioactive peptides derived from vegetable proteins are not found in large quantities. However, they have been studied due to their biofunctional effects and are excellent candidates for developing new products for healthcare and functional food [95].

The harvesting and processing of vegetable food generate a significant amount of residue and by-products that sometimes have excellent quality and may be used to isolate bioactive peptides. In the case of fruit, at least 25 to 30% is disposed of as residue during processing; this mainly consists of peels, pomace, and seeds, which may contain high-quality protein that can release bioactive peptides upon hydrolysis [96]. In Table 4, some residues and subproducts from plant origin are shown, from which peptides have been isolated, classified by the type of residue, such as (1) from grains, seeds, and oilseeds, (2) from fruit, and (3) from vegetables and sub-utilized parts.

In the residue generated by grains such as rice and wheat, the peptides were isolated from the bran protein. In the case of Ren et al. [97], who hydrolyzed rice bran with three different proteases (pepsin, trypsin, and alcalase), they obtained a better antioxidant activity (ORAC and DPPH) in vitro with the fraction <1 kDa of the hydrolyzed rice bran with trypsin. Meanwhile, Kumar et al. [98] demonstrated the best antioxidant activity with the fraction of <3 kDa from the hydrolyzed protein using alcalase; this same fraction also exhibited the best in vitro inhibition of the Angiotensin-Converting Enzyme (ACE). The enzyme used in protein hydrolysis significantly impacts the physical and chemical characteristics of the resulting peptides. However, other factors, such as the hydrolysis conditions and the fractions used in the trials, also play a role.

On the other hand, Zou et al. [99] inhibited ACE in vitro with a fraction of <1 kDa from the hydrolyzed wheat bran; this same fraction resulted in the most significant decrease in systolic arterial pressure in rats with spontaneous hypertension (SHRs). The peptides of the other by-products or residues, such as pumpkin oil cake, black sesame cake, and palm kernel cake, exhibit the inhibitory activity of ACE in vitro, as well as DPP-IV inhibition related to hypoglycemic activity [100,101,102]. Chaipoot et al. [100] and Prados et al. [103] reported that peptides from olive seeds and black sesame, respectively, have hypocholesterolemic effects.

Additionally, residues from fruits and vegetables, such as seeds, peels, and underutilized parts, serve as important sources of protein. Wen et al. [104] obtained peptides from the protein of watermelon seeds hydrolyzed with alcalase; the fraction with a molecular weight of less than 1 kDa exhibited an antioxidant and cytoprotective effect in vitro in HepG2 cells. They identified five peptides, as shown in Table 4, from which the RDPEER peptide, with low molecular weight, containing amino acids and exhibiting hydrophobicity, demonstrated antioxidant activity and cytoprotection against oxidative damage, as well as an increase in the activity of antioxidant enzymes. In the case of Meshginfar et al. [104] and Meshginfar et al. [105], they hydrolyzed the tomato seed protein, and the resulting hydrolysate showed antioxidant capacity, calcium-binding ability, and ACE inhibition. It was found that hydrolyzed peptides with a better capacity for calcium binding had a greater molecular weight (24–80 kDa) than those with better antioxidant capacity and ACE inhibition, which requires extensive hydrolysis to obtain low-molecular-weight peptides. The protein from the peach seed is another residue of the fruit that has been the subject of various research studies. Hernández-Corroto et al. [106] obtained a hydrolysate with alcalase that exhibits antioxidant and antiproliferative activity in the cellular lines HeLa, PC-3, and HT-29, with a more significant effect on the latter.

Hydrolyzed from the underutilized parts of vegetables, such as cauliflower, broccoli, cabbage, and beetroot leaves [107], as well as moringa leaves [108], also showed antioxidant activity and reduced production of inflammatory biomarkers. A significant number of research studies have been conducted using vegetable residues or by-products to minimize environmental pollution, maximize the utilization of plant products, and search for natural compounds with biological activities that benefit human health. The plant-origin peptides have experienced a significant surge in popularity due to their natural origin, characterized by high proportions of hydrophobic amino acids, which confer excellent functionality. Unlike those of animal origin, they are more affordable and widely distributed in nature.

**Table 4:** Peptides from vegetable by-products.

By-Product	Process	Fraction/Peptide/Sequence	Bioactivity/Assays	Reference
**Grain, seed, and oilseed**				
Pumpkin (*Cucurbita maxima*) oil cake	Hydrolysis 1. Pepsin 2. papain	Fraction Hydrolysates and <1 kDa	ACE inhibition and DPP-IV inhibition activity/in vitro	[101]
Pumpkin oil cake	In silico	IAF (Ile-Ala-Phe)	Antihypertensive/in silico	[109]
Olive seed	Hydrolysis with alcalase (AH) and with papain (PH)	Hydrolysates (AH y PH)	Antioxidant (Caco-2 Cells)/in vitro Inhibits DPP-IV (Caco-2 Cells)/in vitro	[110] [111]
Olive seeds	Hydrolysis with alcalase	ADLY, FLPH, KLPLL, and TLVY	Anticholesteremic (HEPG-2)/In vivo	[103]
Olive seeds Genotypes (Manzanilla, Gordal, Verdiel, Cornicabra, and Lechin)	Hydrolysis with alcalase	Hydrolysate (Identified Peptides: KLPLL, WSPLNN, TLPLL, ALMSPH, FVVLK, SSPLL, KLGNF, SHTLVY, VVVVPH, VVLK, ALMAPH, HTLY, VFDGE, FLPH, TLVY, WSMH, QGDLL, WNVN)	Antioxidant Antiproliferative (Cel. HeLa, PC-3, and HT-29)/In vitro	[106]
Black sesame cake	Flavourzyme	Fractions <3, 3–10, and >10 kDa	Inhibitor of DPP-IV, ACE, α-amylase, α-glucosidase, and pancreatic lipase/In vitro	[100]
Rice bran	Hydrolysis with Trypsin	Hydrolysates (Identified Peptide: AFDEGPWPK (1045.48 Da))	Antioxidant (ORAC and DPPH)/In vitro	[97]
Rice bran	Hydrolysis with alcalase, pepsin, and trypsin.	Fraction <3 KDa (alcalase) (Identified Peptides: STCCK KICILVFTLTTC and FMKSK)	ACE inhibition and antioxidant activity/In vitro	[98]
Wheat bran	Hydrolysis with alcalase	Fraction <1 kDa	Antioxidant, renin inhibition, and angiotensin-converting enzyme (ACE) inhibition/in vitro and systolic blood pressure decrease in vivo	[99]
Palm kernel cake	Hydrolysis with papain	YGIKVGYAIP, GGIF (substrate-type), and GIFE (true-inhibitor type)	angiotensin-converting enzyme (ACE) inhibition	[102]
**Fruits’ by-products**				
Watermelon seeds	Hydrolysis with alcalase	Fraction <1 kDa (Identified Peptides: RDPEER, KELEEK, DAAGRLQE, LDDDGRL, and GFAGDDAPRA	Antioxidant Cytoprotective effects/in vitro (HepG2 cells)	[104]
Tomato seeds	Hydrolysis with alcalase	Hydrolysates	Antioxidant, calcium binding, and angiotensin-converting enzyme (ACE) inhibition	[105]
Peach seeds	Hydrolysis with alcalase Genotype Campiel	Hydrolysates Identified Peptides (LVAVSLL, LVDGF, VELT, YQLS)	Antioxidant Antiproliferative (HeLa, PC-3, and HT-29 Cells)	[106]
**Plant waste or underutilized parts**				
Cauliflower, broccoli, cabbage, beetroot leaves	Hydrolysis with pepsin and pancreatin (GSD)	Hydrolysates	Antioxidant	[107]
Moringa (leaves)	Hydrolysis with pepsin and pancreatin (GSD)	Hydrolysates	Antioxidant Anti-inflammatory	[108]

### 4.3. Peptides from Marine By-Products

**Figure 2:**
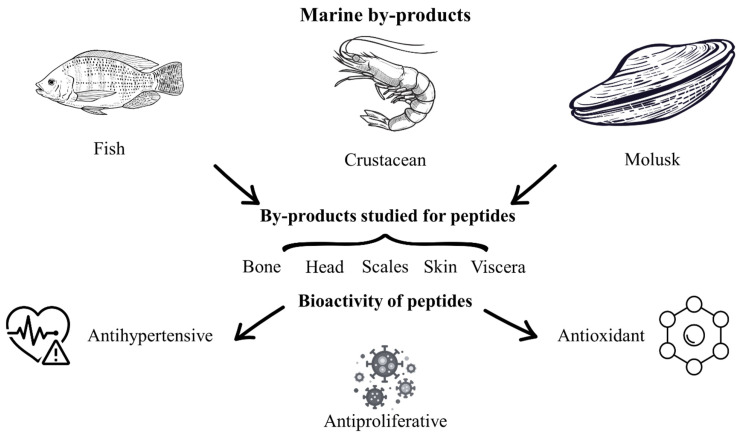
Marine by-products studied for peptide extraction and their bioactivities.

Marine resources constitute a highly diverse source of food, encompassing algae, crustaceans, shellfish, mollusks, and fish [28,112]. During seafood processing, approximately 50% of the weight is discarded as by-product, which poses environmental problems because these marine by-products are traditionally disposed of in the environment and are subject to rapid decomposition, resulting in foul odors and destabilization [28]. Marine by-products comprise backbones, dark muscle, heads, skin, viscera, and non-comestible parts of crustaceans, mollusks, and fishes (Figure 2) [28,113]. Different investigations have been conducted on marine by-products to valorize and reduce the environmental problems. In this sense, marine by-products have been used to obtain bioactive peptides through chemical, enzymatic [113], physical, and emerging methods [29,43,53,112]. Peptides obtained from marine by-products such as shrimp, salmon, sardine, tuna, horse mackerel, and other by-products have important bioactivities such as antibacterial, anti-diabetic, anti-inflammatory, antihypertensive, anti-allergenic [114], antioxidant [24,28], antimicrobial [115,116], and antiproliferative activities [113]. In this context, it has been reported that marine by-products require additional steps to obtain peptides compared to other food sources, due to their complex structure and the limited standardization of the extraction process [117].

In general, the bioactive peptides have been extracted by hydrolysis and/or extraction processes (enzymatic hydrolysis, fermentation, ultrasound-assisted extraction or microwave assisted-extraction), later a purification and analysis steps (combination of membrane filtration such as ultrafiltration or nanofiltration, and chromatographic techniques such as Size-Exclusion Chromatography, reverse-phase High-Performance Liquid Chromatography) are carried out before the composition and peptides structure studies (amino acid analysis by cation exchange or post-column derivatized amino acid) have been done [29,36,117]. For the enzymatic hydrolysis, it has been reported that the enzymes used for peptides extraction from different fish, squid, and other marine by-products are alcalase, bromelain, corolase, chymotrypsin, flavourzyme, proteinase K, thermolysin, pepsin, neutrase, and papain, which could be obtained from animals, plants, or microorganisms [29,117,118,119].

Rodríguez-Jiménez et al. [114] reported that bromelain, pepsin, thermolysin, alcalase, and protamex enzymes have been widely studied for the production of bioactive peptides from fish, mollusks, and shrimp waste. On the other hand, another recent process is fermentation, where pH, temperature, and the inoculum (mainly bacteria and fungi) must be optimized to obtain the highest purity. In this regard, Martí-Quijal et al. [24] reported that *Lactiplantibacillus plantarum*, *Aspergillus oryzae*, *Pediococcus acidilactici*, and *Enterococcus faecium*, among other microorganisms, have been studied on fish, shellfish, and shrimp by-products. Even the marine by-products have been studied to obtain bioactive compounds such as carotenoids, fatty acids, polysaccharides, among others, the bioactive peptides extraction from these by-products is on a rise, due to the higher amount of waste generated on the fisheries and the industry, however; the appropriate method to obtain these bioactive compounds is still under development [117,120].

According to the reports presented in Table 3, Table 4, and Section 4.3, which focus on peptides of animal, plant, and marine origin, respectively, distinct functional profiles have been identified. Peptides from animal by-products exhibit functional dominance in inhibiting ACE and displaying antioxidant activity. Additionally, they possess antimicrobial potential, especially those isolated from bovine hemoglobin. These peptides are, in general, rich in amino acids such as Proline and Glycine, which are commonly found in structural proteins and have a direct influence on the inhibitory activity of ACE. In the case of plant-origin peptides, their functional dominance centers on metabolic inhibition, with an apparent effect on the enzymatic inhibition of ACE and DPP-IV/Glucosidase. These compounds are rich in hydrophobic amino acids (such as Leucine, Valine, and Phenylalanine), which correlate with a greater enzymatic affinity and a marked antioxidant effect. For their part, marine-origin peptides offer a wider range of functionalities, primarily centered on immunological modulation (with anti-inflammatory and anti-allergenic activity) and antimicrobial defense. This combination has high potential for applications in managing chronic responses and restoring homeostasis.

Regarding applications, animal-origin peptides are ideal for the development of antihypertensive and antioxidant nutraceuticals, thanks to their richness in proline and glycine and their ACE inhibitory activity. Plant-origin peptides are the strongest candidates for functional foods focused on cardiometabolic management, due to their double enzymatic inhibition (ACE/DPP-IV). Ultimately, marine-origin peptides stand out for their broad immunological modulation and antimicrobial activity, suggesting a greater potential for the development of biomedical or pharmacological agents to control chronic inflammation and pathogens.

## 5. Purification and Identification of Peptide By-Products

Bioactive peptides are polymers with a low molecular weight, consisting of 2 to 20 amino acids [59]. Therefore, different unit operations are necessary to obtain and isolate peptides, such as hydrolysis, fractionation, purification, and identification, since they are encrypted in protein structure [79,121]. After the hydrolysis process, the protein fraction contains many interfering compounds, including pigments, lipids, phenolic compounds, proteolytic and oxidative enzymes, carbohydrates, and nucleic acids, which must be separated using methods such as ultrafiltration, ion-exchange chromatography, electrophoresis, and reverse-phase high-performance liquid chromatography (RP-HPLC). All purification methods are related to the chemical and physical characteristics of the peptides [122]. Finally, the identification of the peptides by ESI-MS, MALDI-TOF, LC-MS/MS, LC-Q-TOF, and in silico analysis (Table 5) [123,124,125].

### 5.1. Ultrafiltration

This technique is based on membrane filtration and is also an economical, fast, and easy process [126,127]. In the process, different cut-off sizes are used to separate peptides with molecular weights ranging from 1 to 10 kDa. In the ultrafiltration method using a porous membrane, it is possible to selectively retain peptides and eliminate smaller compounds, such as water, salts, and residues, which permeate through the membrane. This method is typically used for concentration, but different washes and fractionations can also be performed. To develop this process, ultrafiltration centrifuge tubes (Amicon Ultra), Macrosep Advance Centrifugal devices, cassette-type membranes, and a QuixStand ultrafiltration system are used.

Additionally, different membrane types, including cellulose, polysulfone, polyether sulfone (PES), cellulose acetate (CA), and Teflon, are employed. However, some disadvantages exist, like possible interactions of peptides with the membrane, saturation, or membrane fouling [128,129,130]. Ultrafiltration is also utilized in electrodialysis technology, enabling the separation of molecules based on their electrical charge and molecular mass. Therefore, Doyen et al. [131] evaluated the impact of ultrafiltration membranes (CA and PES) on peptide migration from snow crab by-product in electrodialysis, which was hydrolyzed with Protamex, a proteolytic enzyme blend. They reported no difference between ultrafiltration with PES and CA of snow crab by-product hydrolysate. The electrodialysis system allows peptide migration without membrane interaction, regardless of the material. Another study involved using membrane ultrafiltration (Amicon cellulose membrane) to separate fractions from the hydrolysates of yellow tuna (*Thunnus albacares*) viscera. Four fractions were reported to contain amino acids, cations, and hydrophobic compounds associated with antioxidant and antibacterial activity [132]. Using different membranes in a sequential configuration enables better fractionation and purification of peptides, while also reducing fouling.

### 5.2. Mass Spectrometry (MS)

It is a widely used technique for separating electrically charged compounds in a gaseous state. These charged compounds, known as ions, are generated by an ionization system or ion source. Then, ions are transferred into the mass detector for identification based on the mass-to-charge ratios (*m*/*z*) [133]. In this sense, the MS is the technique preferred for identifying and probing the structure of proteins [134].

In the peptidomics technique, mass spectrometry is essential for improving peptide visualization and identification [135]. In this sense, the most widely reported spectrometric technologies are tandem mass spectrometry by MALDI-MS/MS (matrix-assisted laser desorption/ionization tandem mass spectrometry) and ESI-MS (electrospray ionization mass spectrometry). The acquisition is a crucial factor in bottom-up proteomics; the most commonly used mode of acquisition is data-dependent acquisition (DDA), although data-independent acquisition (DIA) is another option. DDA acquires subsets of precursor ions, typically the most abundant top-N in the spectrum, but is constrained by the speed of the equipment. DDA, coupled with the dynamic exclusion method, increases the proteome’s detection range. For its part, DIA is the systematic acquisition of MS/MS spectra, independent of the content of MS spectra. Once obtained, the spectra are analyzed using various software, such as Mascot, Comet, SEQUEST, X Tandem, MS-GF+, and MaxQuant [136].

### 5.3. In Silico

These methods utilize theoretical computer or bioinformatics tools to correlate physical, chemical, and mathematical parameters in the study of biomolecules and their therapeutic targets. They also provide the advantages of requiring less time and investment. An in silico tool has become a strategy for peptide design, generating predictions based on a simulated panorama of their biological activity [137]. Therefore, databases and software have been developed to predict structures and interactions with target protein complexes, enabling the discovery of their bioactivity [138]. The evaluation of biological activity by in silico analysis represents an alternative that is currently being widely studied to predict the release of peptides by multi-enzyme screening, as well as to know their bioactivity (antioxidant, anticancer, antitumor, antidiabetic, anticoagulant, antihypertensive, anti-inflammatory, and antimicrobial), and their bioavailability [139]. In silico analyses also include homology modeling, 3D peptide design, molecular dynamics, protein docking, and protein–protein interaction; all these can be developed with algorithms, tools, and computational software [138]. In this context, BIOPEP-UWM, formerly known as BIOPEP, is a database of bioactive peptides derived from food, which many researchers utilize due to its free accessibility. This database is constantly updated, with new peptides or new activities of existing peptides being added [140]. Likewise, Senadheera et al. [141] developed in silico research about cucumber sea by-products (flowers and internal organs) peptides and their antioxidant and ACE activity. First, the peptide sequence was identified using PepRank (Peptide Ranker), and then the sequences were uploaded to BIOPEP-UWM to determine the antioxidant activity of the peptides. They found 17 dipeptides and tripeptides with ACE inhibitory activity. The peptides GPPGPQWPLDF, APDMAFPR, and GPGMMGP, after simulated digestion, presented a potent ACE inhibition. Similarly, the SwissADME (Absorption, Distribution, Metabolism, and Excretion) tool was used to predict a model for drug development, finding that the dipeptides PL, DF, GP, and AF meet the Lipinski criteria and have the potential to be a source of bioactive peptides for drug development.

**Table 5:** Purified and identified peptides from by-products.

By-Product/ Extraction	Purification/Fractionation	Identification In Vitro	In Silico Tool/Database/ Software	Peptide/Sequence	Activity	Reference
Duck liver fermented using *Bacillus subtilis*	Sephadex G-15 gel filtration chromatography. RP-HPLC: columna C18 (150 mm × 2.1 mm, 130 Å, 2.7 µ) at 40 °C. Elution solution A: 100% acetonitrile, B: 0.1% trifluoroacetic acid. UV wavelength 220 nm	LC-MS/MS.	BIOPEP	MYGAVTPVK, NWEKIR, APGIIPR, RWWQLR	In silico Antithrombotic, antioxidant, ACE, DPP-IV, and renin inhibitory activity. In vitro Protect HepG2 cells from oxidative stress, reduce inflammatory cytokines TNF-α and IL-1β, increase antioxidant enzymes activity (SOD, CAT, and GSH-Px), and inhibit lipid oxidation (MDA)	[88]
Duck plasma/hydrolysis alcalase	Ultrafiltration (10 and 3 kDa molecular weight cutoffs), size-exclusion chromatography (Sephadex G-25 column), and HPLC system with phase reverse column (300SB-C18 4.6 mm × 250 mm).	Nano-LC-MS/MS, Hypersil Gold C18 column (2.1 mm × 150 mm, 1.7 μm)	Not evaluated	LDGP, TGVGTK, EVGK, RCLQ, LHDVK, KLGA, AGGVPAG	In vitro Antioxidant capacity: Reduce ABTS and DPPH activity	[142]
Smooth-hound (*M. mustelus*) fish viscera/hydrolysis with Esperase (protease from *Bacillus* sp.)	Ultrafiltration membrane (Amicon MWCO of 50 kDa and 5 kDa), RP-HPLC, C18 column (250 mm × 4.6 mm)	NanoESI-LC-MS/MS, nano HPLC capillary column C18 (3 μm, 75 μm × 12.3 cm)	BIOPEP	IAGPPGSAGPAG, VVPFEGAV, PLPKRE, PTVPKRPSPT	In silico ACE inhibitory activity, antioxidant, and anti-thrombotic In vitro Strong ACE inhibitory activity In vivo Blood-lowering in hypertensive rats	[143]
Milk permeate	Fractionation by solid-phase extraction with a STRATA C18 E cartridge. RP-HPLC, SNAP KP-C18-HS 12 g column (particle size 50 μm, surface area 400 m^2^/g, pore volume 0.95 mL/g, 90 Å pore diameter.	LC-MS/MS, column XB-C18 Aeris Peptide 3.6 μm	Mascot Search engine, UnitProt database	Sequences derive from α and β caseins, lactoperoxidase, lipoprotein lipase, glycosylation-dependent cell adhesion molecule 1, and polymeric immunoglobulin Receptor.	In vitro Protect against oxidative stress by Keap1/Nrf2 activation and antioxidant enzymes In vivo Protect a zebrafish model from cold stress.	[91]
Bovine blood/hydrolysis with alcalase, netrase, and papain.	Ultrafiltration membrane (Amicon MWCO 3–10 kDa, size exclusión chromatography (Superdex peptide 10/300 GL column UV at wavelength 215 nm	LC-MS/MS, C18 column (15 cm × 75 µm with a particle size of 3 µm)	DFBP program	IAWGK, VDLL, MTTPNK, VEDVK, MPLVR, TVIL, KIII, LPQL, DFPGLQ	In silico Antioxidant activity, ACE, and DPP-IV inhibitory In vitro Antioxidant activity: ABTS, DPPH, and metal chelating activity	[144]
Bovine hemoglobin/ hydrolysis with pepsin	Ultrafiltration membrane (regenerated cellulose, MWCO 1–3 kg.mol^−1^). Size exclusion chromatography, column Superdex peptide HR10/3000 (10 mm × 300 mm)	RP-HPLC/MS, column C18 Prophere (250 mm × 21 mm, 5 µ diameter beads)	Not evaluated	Neokyotorphin (NKY) peptide	In vitro High antimicrobial activity against *Micrococcus gluteus*, *Listeria innocua*, *Escherichia coli*, and *Salmonella enteritis*.	[94]
Pectoral fin from Salmon/hydrolysis with pepsin	To fractionate, Sephadex G-25 gel permeation chromatography column (2.7 cm × 98 cm). UV at wavelength 280 nm. The RP-HPLC column Hypersil Gold C18 (20 mm × 250 mm, 5 µm.	Hybrid Quadrupole-TOF LC-MS/MS coupled with ESI source	Not evaluated	Pro-Ala-Tyr (PAY)	In vitro Anti-inflammatory activity, by inhibition of NO/iNOS and PGE_2_/COX-2 pathway, besides inhibiting TNF-α, IL-6 and IL-1β	[136]
Yellow tuna (*Thunnus albacores*) viscera/hydrolysis with Protamex	Sequential ultrafiltration membranes (cellulose) by Amicon (molecular weight cutoff of 3, 10, and 30 kDa)	HPLC, column Ultrasphere ODS 5 μm particle size, 4.6 mm × 25 cm.	Not evaluated	Peptides <3 kDa with cationic and hydrophobic amino acids	In vitro High antibacterial activity against *Listeria*, *Staphylococcus*, *E. coli*, and *Pseudomonas.* Potent antioxidant activity: DPPH, ABTS, and ferric reducing antioxidant.	[132]
Sorghum spent grain/hydrolysis with Neutral protease-Purazyme and Flavourzyme	Sephadex G-25 molecular exclusión, column (20 cm × 0.8 cm). UV at 220 and 280 nm	LC-ESI-Q-TOF	PepDraw PepRank BIOPEP-UVW	GGAAGGR, PPPGSKSYGT, AGLPTEEKPPLL, QADPKTFYGLM, GPPKVAPGK, DISASFGGEWL	In silico Antioxidant, DPP-IV inhibitor. In vitro Antimicrobial: growth inhibition of *Bacillus cereus*. Antioxidant: ABTS scavenging (2.27 mg/mL). Antidiabetogenic (DPP-IV inhibition) IC_50_ 5.49 mg/mL.	[145]
Seed tomato fermented using *Bacillus subtilis*	Size-exclusion chromatography Sephadex G-50 column. UV spectrophotometer at wavelength 214, 254, and 280 nm	HPLC with a Pico Tag column (300 mm × 4 mm, 5 µm). MALDI-ToF/ToF	Not evaluated	Peptides with 500–850 Da and 1200–1500 Da of molecular weight	In vitro Antioxidant and ACE inhibitory activity	[146]
Cauliflower leaves hydrolysis with trypsin	First purification in a solid phase C18 cartridges, then fractionated by a PLR-S column (4.6 mm × 250 mm, 5 µm) in an RP-HPLC at 214 nm, finally purified through an HILIC column (100 mm × 2.1 mm, 2.6 µm) in a RP-HPLC at 214 nm.	NanoHPLC-MS/MS with a 300 m ID × 5 mm Acclaim PepMap 100 C18 (5 μm particle size, 100 Å pore size) pre-column	PeptideRanker	FFAPYAPNFPFK, GGPVPAPCCAGVSK, ILYDFCFLR,	In silico *e* in vitro: ACE inhibitory activity	[147]
Cherry seed/hydrolysis with alcalase, thermolysin, and flavourzyme	SDS-PAGE and RP-HPLC (Poros R2/10 Perfusion column 4.6 mm D × 50 mm) with fluorescence detector at λ_exc_ 280 and λ_em_ 360 nm. Then, a hydrolysis was performed, and a fractionation by ultrafiltration.	HPLC-Q-TOF-MS, ES-C18 (100 mm × 2.1 mm, 2.7 μm particle size, 160 Å pore size) column	Not evaluated	Peptides of molecular weights below 5 kDa (Mw < 1.3 kDa)	In vitro: Antioxidant capacity	[148]
Corn silk/hydrolysis trypsin	Purified by ultrafiltration (membrane molecular weight cut-offs (MWCO) 3 kDa), gel filtration chromatography (Sephadex-G25 column 1.6 cm × 70 cm at 280 nm), and strong-cation-exchange solid-phase extraction (STRATA SCX cartridge)	LC-MS/MS	PEP-FOLD 3.5 AnOxPePred	29 peptides, MCFHHHFHK, VHFNKGKKR, PVVWAAKR, NDGPSR	In silico: Antioxidant Inhibitors of Keap1-Nrf2 interaction, myeloperoxidase, and xanthine oxidase. No toxic, no allergenic, and cell-penetrating potential. In vitro: Antioxidant capacity	[149]
*Asparagus officinalis* L./hydrolysis with alcalase	HPLC connected to Xbridge BEH preparative C18 5 μm OBD 19 × 250 mm	NanoHPLC-MS/MS, 25 cm long fused silica nanocolumn, 75 μm ID, and a precolumn PepMap 100 C18 (5 μm particle size and 100 Å pore size).	Not evaluated	PDWFLLL, FAPVPFDF, MLLFPM, FIARNFLLGW, ASQSIWLPGWL.	In vitro: ACE inhibitory activity	[150]

## 6. Biological Activities of Peptides from By-Products of Plant, Animal, and Marine Origin

**Figure 3:**
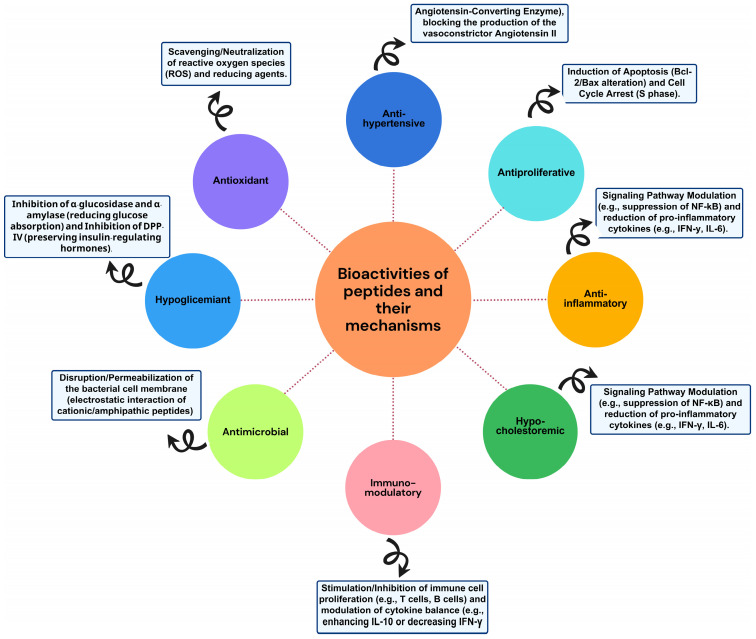
Bioactivities and mechanism of peptides from waste and by-products.

Peptides exhibit diverse biological activities (Figure 3) that can have a positive impact on human health. Below, we describe some of the most studied activities in peptides derived from residues and by-products.

### 6.1. Antioxidant

Antioxidants are a group of compounds capable of counteracting cellular oxidation by acting as quenchers of radical species, reducing agents, scavengers of free radicals, and other pro-oxidants such as metals [151]. By-products of plant, animal, and marine origin are an interesting source of antioxidant peptides. Bioactive peptides from by-products are significant due to their role in preventing oxidative stress, a phenomenon that has been widely documented. To illustrate, cherry seed hydrolysates (55% degree of hydrolysis obtained using thermolysin) inhibited lipid peroxidation and the hydroxyl radical (OH) in 80% and 30%, respectively [148]. Also, jackfruit seed hydrolysates prepared with trypsin showed a radical scavenging activity determined by ABTS^+^ (2,2′-azino-bis(3-ethylbenzothiazoline-6-sulfonic acid) with EC_50_ values of 2.84 mg/mL [152]. Olive seed hydrolysates obtained with alcalase (70.4% degree of hydrolysis) demonstrated to possess the antioxidant capacity of inhibiting lipid peroxidation (92.1%), hydroxyl radical (54.4%), for ABTS^+^ (72.9%), and DPPH (2,2-diphenyl-1-picrylhydrazyl) (68.6%) [153]. On the animal and marine origin by-products side, bovine lungs were hydrolyzed with alcalase, papain, and pepsin [154]. FRAP (Ferric Reducing Antioxidant Power) assay showed low ferric-reducing activity power in the lung hydrolysates. In contrast, in ORAC (Oxygen Radical Absorbance Capacity) determination, the three hydrolysates exhibited values between 400 and 450 µmol Trolox equivalents. Trypsin was used to obtain hydrolysates from porcine liver [155], demonstrating the highest antioxidant activity in ABTS radical scavenging (86.8%). The antioxidant activity of protein hydrolysate of stripped weak fish by-product obtained with Protamex had lower DPPH activity (50–60%) than that obtained with alcalase (60–70%) [156]. After 30 min of hydrolysis of catfish skin using collagenase, the highest values for DPPH inhibition were found (72.5%) [157]. All these studies are in vitro assays.

### 6.2. Immunomodulatory and Anti-Inflammatory

Prolonged inflammation is recognized to be one of the causes of diverse conditions such as arthritis, neurodegenerative and cardiovascular diseases, diabetes, and cancer [158]. Bioactive peptides have demonstrated anti-inflammatory activity, highlighting their beneficial role in managing certain chronic diseases [159]. The capacity of peptides to modify the expression of several pro-inflammatory molecules, such as cytokines, cyclooxygenase, nitric oxide synthase, and lipoxygenase, contributes to the regulation of inflammatory signaling [160,161]. The combination of Corolase PP, Flavourzyme, and Alcalase 2.4 L was utilized for the hydrolysis of brewers’ spent grain protein, generating hydrolysates that considerably decreased pro-inflammatory cytokine IFN-γ production in Con-A-stimulated Jurkat T cells [160]. O’Sullivan et al. [154] reported that hydrolysates of bovine lungs produced with alcalase revealed significant anti-inflammatory activity in RAW264.7 macrophages, showing a substantial reduction in IL-6, IL-1β, and nitric oxide production. Yak bone hydrolyzed by a combination of alcalase, neutrase, and flavorzyme generates peptides GPAGPSGPAGK and GPSGPQGIR, which effectively control the NF-κB signaling pathway and nitric oxide generation to suppress the inflammatory response [161].

### 6.3. Antihypertensive

Hypertension is defined as a long-term elevation of blood pressure above 140/90 mmHg, which can often be improved by adopting healthy lifestyle choices, including dietary modifications, stress reduction, and increased physical activity [162]. Several treatments are available for the management of hypertension. Still, their side effects have led to increasing interest in research into food-derived bioactive compounds for the treatment of hypertension, including bioactive peptides. Different studies have reported that the enzymatic hydrolysis of proteins from different food sub-products is a rich source of protein hydrolysates and bioactive peptides with anti-hypertensive characteristics. These by-products include fruit seed proteins, vegetable leaves, stems, and processed meat by-products. Food by-products have been evaluated in vivo to identify peptides with antihypertensive activity utilizing spontaneously hypertensive rats (SHR). The peptide IYSPH obtained by hydrolysis of peach seed proteins can reduce 30 mmHg of the systolic blood pressure in SHR after 6 h of treatment [163]. Two active peptides, Gly-Leu-Phe-Phe and Leu-Gly-Phe-Phe, obtained from moringa leaf protein hydrolysate, exhibited dual inhibitory activity of ACE (IC_50_ = 0.31 mM, respectively), and SHR reduces systolic blood pressure (19.4 and 18.2 mmHg) and diastolic blood pressure (12 and 13.8 mmHg), respectively [164].

### 6.4. Antiproliferative or Anticancer

Cancer is one of the leading causes of death worldwide, where uncontrolled growth and proliferation of the cells, causing a rapid increase in tissue mass in the affected parts of the body, is caused by a series of genomic/molecular alterations. Anticancer therapies such as chemotherapy and radiotherapy are associated with undesirable side effects because they are unable to differentiate between normal and cancerous cells, in addition to low specificity and the generation of toxicity [165].

Dia and Krishnan [166] identified and isolated the BG-4 peptide from *Momordica charantia* seeds, which causes cytotoxicity to HT-29 and HCT-116 and human colon cancer cells (IC_50_ 217 and 134.4 μg/mL, respectively) due to the reduction in the expression of Bcl-2 and increasing the expression of Bax, affecting the expression of cell cycle proteins CDK2 and p21.

KPEGMDPPLSEPEDRRDGAAGPK and KLPPLLLAKLLMSGKLLAEPCTGR, two peptides identified in tuna cooking juice hydrolysates by protease, showed antiproliferative effect, induced cell cycle arrest in S phase, and apoptosis against MCF-7 cells by downregulation of the expression of Bcl-2, caspase 9, and PARP without affecting normal breast epithelial cells [167]. Lu et al. [168] reported the activity of two peptides from cod skin gelatin hydrolysates (GEIGPSGGRGKPGKDGDAGPK and GFSGLDGAKGD), prepared with alkaline protease and trypsin, that have essential actions in different invasive processes, inhibiting MMP-1, p-p38, and p-ERK. Peptide LLPSY obtained by hydrolysis of olive seed proteins showed an antiproliferative effect on breast cancer cells (MDA-MB-468) and prostate cancer cells (PC-3) and showed the capability to modify cell adhesion, arrest cell cycle in S phase, and decrease the migration capacity of cancer cells [169]. Muster olysin, a peptide obtained from a mustard oil refinery with the sequence NH2-KYQFFVP-COOH, exhibits proapoptotic activity against human epidermoid cancer cells (Hep2), likely due to its capacity for rapid membrane attachment, which may result in cell death [170]. A purified peptide from hydrolysates of walnut residual protein inhibited the cancer cell growth of HeLa and Caco-2 (IC_50_ = 0.60 and 0.65 mg/mL, respectively) without presenting cytotoxicity against IEC-6 cells, a non-cancerous [171].

### 6.5. Antimicrobial

“Antimicrobial agent” is any chemical compound (synthetic or natural), peptide, polypeptide, or nucleic acid that presents and exerts antibacterial, antifungal, antiviral, and/or antiyeast activity. In particular, antimicrobial peptides and proteins (AMPs) have been identified in by-products from animal, marine, and plant origins, as they are active ingredients that could enhance the antimicrobial effect by inhibiting a wide range of microorganisms through various mechanisms, mainly targeting cell membranes or specific intracellular components [172]. Most are amphipathic (hydrophobic and hydrophilic) and cationic (positively charged) peptide molecules. Membrane permeability is primarily recognized as a well-accepted mechanism for describing the action of cationic AMPs. These cationic AMPs present amphipathic type helices that act as a bactericidal structure, which can bind and interact with negatively charged bacterial cell membranes, leading to the change in the electrochemical potential in the bacterial membranes, inducing damage to the morphology and cell membranes, generating the permeation of larger molecules such as proteins, to result in cell death [173]. Overall, the amphipathic helix structural motif is crucial to the antimicrobial activity of these peptides, as it induces bacterial cell destruction.

Various studies have evaluated the antimicrobial activity of hydrolyzed proteins and/or peptides derived from marine by-products, such as fish and/or mollusks. Djellouli et al. [174] obtained peptides from the exoskeleton of shrimp (*Litopenaeus vannamei*) using specific microorganisms such as *Enterococcus faecalis* DM19, isolated from Curda camel milk, which are producers of proteases generated during the fermentation process. The hydrolysates were heated with glucosamine (GlcN) at 100 °C for 0, 40, 60, 120, and 180 min. As a result, they exhibited good antibacterial activity against both Gram-positive and Gram-negative bacteria compared to native hydrolysates without GlcN. Likewise Wald et al. [175] studied the hydrolysates of rainbow trout (Oncorhynchus mykiss) by-products with pepsin. They demonstrated that the degree of hydrolysis exerts a considerable influence on inhibitory activity against Gram-positive and Gram-negative bacteria, such as *Flavobacterium psychrophilum* and *Renibacterium salmoninarum*, with a significant increase in the inhibitory effect observed at the beginning of hydrolysis, reaching 30%. The determination of amino acids of the hydrolysate revealed that lysine, leucine, alanine, arginine, glycine, and glutamic acid residues represented the primary inhibitory amino acids.

On the other hand, there are few known investigations of antimicrobial peptides in plant food by-products. Tan et al. [176] studied palm kernel cake, a beneficial by-product of palm kernel oil production. Their research isolated the by-product peptides with enzymatic hydrolysis using alcalase. The peptides were found to be suitable for use as an antimicrobial agent with potent antibacterial activity, particularly against Gram-positive bacteria, such as *Bacillus* species. Another study carried out by Suarez et al. [177] demonstrated that the Flo peptides from the crushed seed of Moringa oleifera produce damage to the bacterial membrane and that this activity is located in a sequence prone to forming the helix-loop-helix structure, decreasing the viability of Gram-negative and Gram-positive bacteria (*P. aeruginosa*, *Streptococcus pyogenes*, and *S. aureus*), without having toxic effects on red blood cells in humans.

### 6.6. Hypocholesterolemic

Hypercholesterolemia is a metabolic disorder associated with a high risk of cardiovascular diseases. This disorder is characterized by high levels of cholesterol in the blood. There are two sources of cholesterol: endogenous cholesterol, which is synthesized by the body, and exogenous cholesterol, which is obtained through the diet. Likewise, searching for alternative natural compounds, such as bioactive peptides derived from by-products, can significantly reduce endogenous and exogenous cholesterol [103,178]. Most bioactive peptide strategies are based on disrupting micellar cholesterol transport and inhibiting enzymes involved in the absorption and synthesis of cholesterol and triglycerides. One of the most studied mechanisms is the inhibition of the reductase enzyme 3-hydroxy-3-methylglutaryl-coenzyme A (HMG-CoA), which catalyzes the rate-limiting step in this process; therefore, its inhibition is essential for reducing cholesterol biosynthesis in the body.

Previous studies on by-products of plant origin have indicated that peptides generated by the hydrolysis of olive seed proteins exhibit a multifunctional capacity to reduce lipids, both in vitro and in vivo, by inhibiting the HMG-CoA reductase enzyme. Prados et al. [179] found that olive seed peptides hydrolyzed with alcalase were the best enzymatic option for this purpose. Through their characterization and identification by RP-HPLC-MS, they demonstrated that the NFVVLK peptide was correlated with the ability to reduce the absorption of exogenous cholesterol. In contrast, the WVAF peptide was associated with lowering endogenous cholesterol. Subsequent studies inhibited endogenous cholesterol synthesis by inhibiting the enzyme HMG-CoA reductase, resulting in a 38.7% inhibition. Two in vivo trials were conducted using a low concentration of hydrolysate (200 mg/kg/day) in male and female mice, resulting in a 25% reduction in total cholesterol levels. The analysis of olive seed hydrolysate using reversed-phase high-performance liquid chromatography and mass spectrometry (RP-HPLC-MS) allowed the identification of peptides that could be responsible for this hypolipidemic effect [103].

Recently, researchers have reported the importance of hydrolyzed peptides from marine by-products in controlling cholesterol metabolism [180]. Karoud et al. [181] evaluated the potential of protein hydrolysates from hake heads (*M. merlucio*) in male Wistar rats with hypercholesterolemia induced with a high-cholesterol diet during six weeks of treatment. The high cholesterol diet caused dyslipidemias, and the addition of hake protein hydrolysates attenuated these abnormalities in a dose-dependent manner.

### 6.7. Opioid Activity

Unlike some studies that have analyzed opioid activity in hydrolysates of proteins and/or peptides of animal and plant origin [182], to our knowledge, there are very few reports on the presence of this bioactivity in hydrolysates or peptides derived from by-products. To consider that they have opioid activity, peptides from exogenous sources have demonstrated analgesic effects and exhibit a good affinity for the opioid receptor, a transmembrane protein in brain regions, allowing them to exert hormonal and neuromodulatory effects similar to opiates by influencing the nervous system and gastrointestinal functions [183]. Peptides from exogenous sources are called exorphins. Its main advantages are stability and fewer side effects compared to endogenous and synthetic opioid peptides. In general, the physiological regulatory activity of opioid peptides encompasses the regulation of gastrointestinal motility, pancreatic secretion, gastric digestion, and the relief of stress and pain [184].

Görgüç et al. [3] reported that typical opioid peptides consist of three types of precursor proteins: proopiomelanocortin (endorphins), proenkephalin (enkephalin), and prodynorphin (dynorphins). They are generally characterized by containing Tyr-Gly-Gly-Phe at their N-terminus, and, in the case of atypical opioid peptides, a Tyr residue is found at the N-terminus [185]. These peptides have been reported to behave like morphine in the brain and are biologically very potent. Potentially, micromolar amounts may be sufficient to exert physiological effects [186].

Kaur et al. [187] mentioned that most plant sources contain opioid peptides derived from wheat, corn, oats, rye, barley, and certain vegetables. However, the opioid effect could also be primarily related to the by-products generated from these sources.

## 7. Application of Peptides

Peptides possess properties that influence human health and improve food quality, among other benefits, resulting in a wide range of potential uses. These compounds are beneficial in the development of numerous healthy functional diets; they are used as sweeteners, color stabilizers, thickeners, anti-caking agents, emulsifiers, flavor enhancers, and acidity control. Peptides can also influence the finished product’s water and oil retention capability, colloidal stability, viscosity, and foam generation [188].

Peptides possess health-promoting properties and disease-prevention capabilities, which is why their application as nutraceuticals is as enjoyable as their use in food enhancement. In this sense, many peptides with activities such as ECA inhibitors, antioxidants, antimicrobials, antidiabetics, immunomodulators (including anticancer and anti-inflammatory agents), and anticholesterolemic agents have been identified. These bioactive compounds are formulated in various forms for administration, including powders, capsules, and ingredients integrated into functional foods such as beverages or nutritional bars. An essential challenge in this field is to ensure that peptides maintain their stability and bioactivity upon transit through the gastrointestinal tract, which is dependent on their bioavailability [13].

Beyond food and nutrition, peptides are increasingly explored for therapeutic use. The development of these compounds as pharmaceuticals is complex due to their low oral bioavailability and rapid in vivo degradation. Consequently, a significant portion of the most recent research focuses on sophisticated delivery systems. This includes encapsulation in nanocarriers (such as liposomes or polymeric nanoparticles) to protect them from enzymatic breakdown and target specific tissues. Furthermore, new routes for systemic administration are being explored [125].

The use of peptides as cosmeceuticals is also well known; besides their bioactivity, low toxicity, and hydrating properties, two additional aspects have been recently considered: bioavailability and stability (to light and oxygen). Cosmeceutical peptides can function as signal molecules to stimulate the production of dermal proteins, such as collagen and elastin, or as neurotransmitter inhibitors to reduce muscle contraction and minimize fine lines. These compounds treat skin afflictions as antioxidants and anti-wrinkle agents, promoting collagen synthesis and possessing antioxidant, anti-inflammatory, bleaching, and healing properties [189,190]. Peptides with antimicrobial activity against bacteria such as *S. aureus*, *S. epidermidis*, *E. coli*, and *P. aeruginosa* have been used to heal in vitro and in vivo injuries, as they stimulate re-epithelialization and angiogenesis in skin lesions. They also promote the proliferation of keratinocytes, fibroblasts, and monocytes, all of which are crucial to the healing of scars [191]. Other biomaterials, such as gels, collagen films, and membranes derived from marine collagen, have also demonstrated practical applications in scar healing [192].

Other uses of bioactive peptides include enhancing the nutritional quality and mitigating the anti-nutritional effects associated with the use of plant and animal proteins in animal feed through chemical, enzymatic, or microbiological processes [193].

## 8. Conclusions and Future Perspectives

Although it has been demonstrated that peptides exhibit a wide range of biological activities, particularly those related to human health, their potential as nutraceuticals requires industrial scaling technology to enhance the viability of producing these bioactive compounds. Therefore, the scientific community’s effort is needed to valorize residues, especially those stemming from agro-industrial processes, agriculture, and other underutilized materials, as it has been proven that they are a key protein source and can be utilized under the concept of sustainability and circular economy [76].

This review concludes that their specialized origin must guide the optimal application of bioactive peptides. Peptides from animal by-products are suitable for the development of antioxidant and antihypertensive nutraceuticals. Vegetable by-product derivatives are the best option for cardiometabolic functional foods. Ultimately, marine peptides exhibit immunomodulatory and antimicrobial properties, indicating their potential for the development of pharmacological and biomedical agents to manage chronic inflammation and pathogens.

Challenges persist in translating the demonstrated in vitro and in vivo efficacy of these peptides into standardized applications. Scientifically, it is crucial to address the choice of the most adequate administration systems for peptides in specific application contexts. Furthermore, pre-clinical and clinical studies are urgently needed to fully determine key parameters for human health, including their dose–response relationship, bioavailability, pharmacokinetics, and safety when consumed alongside food.

Moreover, the industrial viability of bioactive peptides depends on regulatory acceptance and profitability. Standardization remains a significant obstacle; reproducible production protocols are essential, particularly for enzymatic hydrolysis conditions and subsequent purification steps, to comply with regulatory bodies and support biological functionality claims. Therefore, future research must incorporate technical–economic analysis to ensure that the valorization of protein-rich by-products remains economically competitive against established synthetic or naturally sourced compounds, thus fully complying with the circular economy model.
